# Anxiety and depression in diabetes care: longitudinal associations with health-related quality of life

**DOI:** 10.1038/s41598-020-57647-x

**Published:** 2020-05-20

**Authors:** Xiaona Liu, Juanita Haagsma, Eric Sijbrands, Hanneke Buijks, Laura Boogaard, Johan P. Mackenbach, Vicki Erasmus, Suzanne Polinder

**Affiliations:** 1 Department of Public Health, Erasmus MC, University Medical Center Rotterdam, Rotterdam, the Netherlands; 2 Department of Internal Medicine, Erasmus MC, University Medical Center Rotterdam, Rotterdam, the Netherlands; 3 Department of Medical Psychology and Psychotherapy, Erasmus MC, University Medical Center Rotterdam, Rotterdam, the Netherlands; 4 Department of Infectious Disease Control, Shenzhen Bao’an Center for Disease Control and Prevention, Shenzhen, China

**Keywords:** Depression, Preventive medicine, Epidemiology, Outcomes research, Preclinical research

## Abstract

Anxiety and depression are commonly found in patients with diabetes, but little is known about how the anxiety and depression symptoms of diabetes patients and the health-related quality of life (HRQoL) over time influence each other. Therefore, we conducted a survey among patients with diabetes (T1) and repeated the survey after 3 months (T2). Linear regression models and cross-lagged structural equation models were used to analyze the associations between anxiety and depression symptoms and HRQoL within and across time intervals. Correcting for baseline index and potential confounders, the HRQoL index at T2 reflected the change in anxiety/depression between T1 and T2 more than anxiety/depression at T1 (*P* < 0.05). Similarly, anxiety and depression at T2 reflected the change in the EQ-5D index over time more than the index at baseline (*P* < 0.05). Our longitudinal data fitted well in a cross-lagged model with bi-directional pathways of associations between anxiety and HRQoL, as well as depression and HRQoL, among adult patients with diabetes (x^2^/df = 1.102, *P* = 0.256; CFI = 1.000, RMSEA = 0.030). Our findings support early detection of anxiety and depression, as well as comprehensive efforts improving HRQoL for patients with diabetes.

## Introduction

Diabetes is an increasing health problem, especially in Western countries^1,2^. In 2000, the global prevalence of diabetes for all age groups was estimated at 2.8% whereas the expected prevalence in 2030 is 4.4%^1^. In recent years, health-related quality of life (HRQoL) has become one of the major indicators for the health assessment of diabetes care. Improving diabetics’ HRQoL has been considered an important goal of prevention and treatment. HRQoL incorporates physical, psychological and social functioning dimensions^3^. Despite a growing understanding of clinical and laboratory measures to improve HRQoL for diabetes patients^4,5^, few attempts have been made to rigorously study the relationship between HRQoL and psychological factors, like symptoms of anxiety and depression, in diabetes care.

Anxiety and depression commonly exist and affect patients with diabetes^2,6–10^. A previous meta-analyses on the prevalence of depression in adults with diabetes concluded that the likelihood of depression among people with diabetes doubles those among a general population without diabetes^7^. Furthermore, evidence shows that symptoms of anxiety and depression often remain unrecognized in diabetes care^11^. Anxiety and depression may influence how people assess their objective health status, and impact the course of diabetes such as poor glycemic control and medication non-compliance^12^. Also, given anxiety and/or depression is an important domain of the broad concept of HRQoL^13^, it’s easily understandable that the co-occurrence of anxiety and/or depression in patients with diabetics may be associated with a lower HRQoL compared to patients without the co-occurrence of anxiety and depression.

In spite of demonstrating the negative association between anxiety/depression and HRQoL in diabetes care, most previous studies that have been performed on the association had used a cross-sectional design and it’s impossible to determine the casual relationship due to the cross-sectional nature of the studies^12^. As a result, there is little evidence that supports the relationships between anxiety/depression symptoms and HRQoL over time, not to mention relationships taking into account variation among different clinic and demographic subgroups of diabetes patients. In addition, recent studies suggest that depression is bi-directionally associated with several determinants of HRQoL in diabetes care, such as obesity^14^, metabolic syndrome^15^, sexual dysfunction^16^, and severe hypoglycemic and hyperglycemic events^17^, however, to date it’s unknown whether or not anxiety/depression is bi-directionally associated with the overall HRQoL of diabetes.

Longitudinal data are necessary in order to assess the effect of anxiety/depression on HRQoL over time, or vice versa. The hypothesis in this study is that anxiety and depression are associated bi-directionally with HRQoL of diabetes in time. This would suggest a synchronous association between changes in anxiety/depression and HRQoL in diabetes care, which is perhaps similar to the relationship between fatigue and pain in primary care^18^. For example, current HRQoL of diabetes may directly predict anxiety and depressive symptoms in the future. Alternatively, anxiety and depression symptoms may be a consequence of experiencing different HRQoL in diabetes care over time.

This study aims to explore the prevalence of anxiety and depression symptoms and HRQoL in adult patients with diabetes in the Netherlands, and to assess associations between anxiety/depression and HRQoL in time, taking into account adult patients with different demographic and diabetes characteristics. Past evidence has shown that anxiety and depression are remarkably different having distinctive risks, patterns, and familial associations^19^. We therefore investigate the co-occurrence of anxiety and depression symptoms in diabetes patients separately in this study.

## Methods

This is a prospective observational study, and it is part of an implementation study of screening and treatment of depression in diabetes patients following the guidelines at the Erasmus MC, University Medical Center Rotterdam in the Netherlands. The measurements involve digital questionnaires that were administered at baseline (T1) and 3 months’ follow-up (T2). During the preparation phase, the participating healthcare workers received training from the research team, including instructions on study logistics, use of the online screening program, interpretation of scores, discussion of outcomes and advising on referral to a psychologist if indicated. Study data were transferred and saved at the central database of the medical center throughout the project. The study protocol was approved by the ethics committee at the Erasmus University Medical Center Rotterdam and informed consent was obtained from all participants. All methods were performed in accordance with the relevant guidelines and regulations.

### Patients

Adults (aged >18 years) who were diagnosed with type 1 or type 2 diabetes and visited the diabetes outpatient clinic of the hospital from November 2016 to March 2017 were invited for participation in an online survey, after completing a screening program for detecting their risk of depression. The implementation of the screening program followed Dutch National Guidelines^20^ and consisted of two components: a self-administrative anxiety and depression risk assessment, followed by a personal interview of patients who were assessed with risks for anxiety and/or depression. Those who indicated having difficulties to complete the online survey, due to reasons including no computer access, insufficient computer skills, language barriers and cognitive problems, were assisted by trained nurses with tablets onsite. Patients who completed the online survey received an email with a link after 3 months, asking them to repeat the survey.

### Measures

The self-administered digital questionnaire at T1 and T2 included the following aspects.

#### Demographic and diabetes characteristic

Patients were asked about their gender, age, type of diabetes, years living with diabetes, employment status, education background, and the country of birth of themselves and their parents. Individuals were defined as ethnicity of majority if both of their parents were born in the Netherlands; ethnicity of minority if he/she was born abroad and at least one of the parents was born abroad (1st generation), or if he/she was born in the Netherlands with at least one of the parents born abroad (2nd generation)^21^.

#### Anxiety and depression

The validated Dutch version of the Hospital Anxiety and Depression Scale (HADS) was used to detect anxiety and depression symptoms in diabetes patients^22^. This scale has been validated in many countries and its capacity to detect anxiety and depressive disorders is widely recognized^23^. It consists of 14 items, seven of which relate to anxiety symptoms and seven to depressive symptoms. Each item was scored from 0 to 3, therefore participants can score between 0 to 21 for either anxiety or depression. Scores at the anxiety subscale (HADS-A) and depression subscale (HADS-D) were measured as continuous variables in this study, except for the prevalence of anxiety and depression symptoms. We defined a score of 8 or more on either HADS-A or HADS-D to identify DSM-III anxiety and depression symptoms as suggested by Bjelland and colleagues^24^.

#### Health-related quality of life

The validated Dutch version of the five-level EuroQol five-dimensional questionnaire (EQ-5D-5L), developed by EuroQol Research Foundation, was applied to measure health-related quality of life (HRQoL) on five dimensions, i.e. mobility, self-care, usual activities, pain/discomfort and anxiety/depression^25^. Additionally, cognitive functioning was added as a sixth dimension to the five existing dimensions (EQ-5D + C)^26,27^. Each dimension included five severity levels, i.e. no problems, some problems, moderate problems, severe problems, and extreme problems. Furthermore, the EQ visual analogue scale (VAS) was used to measure respondent’s self-rated health. The score ranges from 0 to 100, indicating the “the worst health you can imagine” to “the best health you can imagine”^28^. An EQ-5D summary index was calculated using scores at EQ-5D and the Dutch tariff established from the Dutch population^13^. All dimensions of EQ-5D + C were measured as categorical variables, while EQ VAS, EQ-5D summary index measured as continuous variables in this study.

### Statistical analyses

Data were analyzed using IBM SPSS Statistics and AMOS version 24.0. Descriptive analyses were conducted for demographical and diabetes characteristics, anxiety and depression subscales (HADS), and all dimensions of the EQ-5D + C. Paired chi-square tests and one-way analysis of variance tests were performed to assess differences between T1 and T2 for those variables. Also, differences in diabetes EQ-5D were compared by identifying if a patient filled in a different level of problems on one or more dimensions at T2 compared to the T1. We investigated the longitudinal associations in three steps. First, we assessed the association between HADS and HRQoL at T1 and T2, respectively. The mean and its 95% confidence interval (CI) of HADS-A and HADS-D were calculated and mapped at each of the five severity levels for the dimensions with anxiety/depression dimension excluded. Univariate linear regression analyses were conducted and mapped using HADS-A and HADS-D scores separately to explain the variation at EQ-5D summary index and EQ VAS score within time intervals. Second, we assessed the association across time intervals in four linear regression models, predicting three outcomes at T2, i.e. EQ-5D summary index (Models 1 and 2), HADS-A (Model 3) and HADS-D (Model 4). In the Model 1 and 2, HADS-A/HADS-D at T1 and change in HADS-A/HADS-D between T1 and T2 were firstly added as main predicators separately, then EQ-5D summary index at T1 was adjusted; In the Model 3 and 4, EQ-5D summary index at T1 and change in the index were added as main predicators separately, then HADS-A/HADS-D at T1 was adjusted. We tested the full models by including both main predictors, outcome at T1, and demographical and diabetes characteristics as predicators. Finally, a cross-lagged panel structural equation modelling (SEM) was used to assess the longitudinal association among anxiety, depression and HRQoL (EQ-5D summary index) at T1 and T2. All variables were modeled as observed variables with error terms included to correct for external factors that may contribute to observed effects. We used a number of fit indices to evaluate the model: chi-square (Χ^2^)/degrees of freedom (df) of 2.00 or below, Comparative Fit Index (CFI) values of 0.90 or above, and root mean square error of approximation (RMSEA) values of 0.06 or below were used as standards of acceptable fit^29^.

## Results

### Demographic and diabetes characteristics

At T1, among 305 patients who completed the screening questions, 131 (42.9%) completed the survey for this study (Table 1). The respondents were aged from 23 to 81 years (mean: 54 years). The majority were male (50.4%), Dutch inhabitants (73.6%), living with type 2 diabetes (58.6%) for over 19 years, with a paid employment (43.2%) or social welfare (including retirement) (40%), had received education lower than the college level (71.5%). At T2, among 131 eligible respondents, 113 (86.3%) patients completed the second survey and 18 (13.7%) were lost to follow-up. No statistically significant differences were found regarding the demographic and diabetes characteristics between T1 and T2 (*P* > 0.05).

**Table 1:** Demographic and diabetes characteristics of patients with diabetes at T1 and T2.

	T1 (*n = *131)			T2 (*n = *113)			*P*-value
	n	%	Mean (SD)	n	%	Mean (SD)	
**Gender**							
Male	66	50.4		57	50.4		1.000
Female	65	49.6		56	49.6		
**Diabetes type**							
Type 1	46	41.4		41	42.7		0.982
Type 2	65	58.6		55	57.3		
**Age** (year)			54.0 (13.1)			54.4 (12.7)	0.776
**Duration of living with diabetes** (year)	19.4 (14.3)			19.9 (14.7)	0.807		
**Employment**							
Paid employment	54	43.2		46	43.4		0.934
Social welfare or retirement	50	40.0		44	41.5		
Unemployment	21	16.8		16	15.1		
**Education level**							
Low (LTS, VMBO, MAVO, VMBO-t)	54	41.5		47	42.0		0.910
Intermediate (MTS, HBS, HAVO, VWO)	39	30.0		31	27.7		
High (HBO, WO)	37	28.5		34	30.4		
**Ethnicity**							
Majority/no migrant	95	73.6		87	78.4		0.566
1^st^ generation migrant	24	18.6		15	13.5		
2^nd^ generation migrant	10	7.8		9	8.1		

Note: LTS, lage technische school; VMBO, voorbereidend middelbaar beroepsonderwijs; MAVO, middelbaar algemeen voortgezet onderwijs; VMBO-t, voorbereidend middelbaar beroepsonderwijs-theoretical; MTS, middelbaar technische school; HBS, hoge burger school; HAVO, hoger algemeen voortgezet onderwijs; VWO, voorbereidend wetenschappelijk onderwijs; HBO, hoger beroeps opleiding; WO, wetenschappelijk onderwijs.

### Prevalence of anxiety, depression and HRQoL

Of all respondents at T1 (Table 2), 36 (27.5%) had anxiety symptoms and 26 (19.8%) had depression symptoms. Twenty (15.3%) patients had both depression and anxiety symptoms. Regarding HRQoL, over half of the diabetes patients reported that they had experienced problems in usual activities (53.4%), pain (74.8%) and cognition (51.9%). The mean EQ VAS score was 70.1 (SD: 20.1) out of 100 (best health), the averaged EQ-5D summary index was 0.73 (SD: 0.27) out of 1 (best HRQoL). Among 113 respondents who filled out the survey both at T1 and T2, 61 (46.6%) reported less problems on the EQ-5D dimensions at T2 while 49 (37.4%) reported more problems on the EQ-5D dimensions at T2. No statistically significant differences were found between T1 and T2 regarding anxiety symptoms, depression symptoms, and all dimensions of HRQoL (*P* > 0.05).

**Table 2:** Hospital Anxiety and Depression Scale (HADS) scores, percentage of reported problems on the European Quality of Life at 5 dimensions (EQ-5D) and extended cognitive dimension, the mean score of patients with diabetes for EQ-5D summary score and index and EQ visual analogue scale (VAS) at T1 and T2.

	T1 (*n = *131)			T2 (*n = *113)			*P*-value
	n	%	Mean (SD)	n	%	Mean (SD)	
**Anxiety and depression**							
HADS – Anxiety (0–21)		5.81 (4.12)			5.66 (4.20)	0.778	
Cut-off score:8	36	27.5		31	27.9		0.938
HADS – Depression (0–21)		4.64 (3.94)			4.80 (4.33)	0.763	
Cut-off score:8	26	19.8		26	23.4		0.500
**EQ-5D-5L**							
Mobility			1.76 (1.01)			1.70 (0.97)	0.678
No problem (1)	73	55.7		63	56.8		0.897
Problems (2–5)	58	44.3		48	43.2		
Self-Care			1.18 (0.58)			1.15 (0.47)	0.760
No problem (1)	116	88.5		97	88.2		1.000
Problems (2–5)	15	11.5		13	11.8		
Usual activities			1.92 (1.07)			1.95 (1.01)	0.773
No problem (1)	61	46.6		46	41.4		0.439
Problems (2–5)	70	53.4		65	58.6		
Pain/discomfort			2.24 (0.99)			2.09 (0.92)	0.219
No problem (1)	33	25.2		32	29.1		0.561
Problems (2–5)	98	74.8		78	70.9		
Anxiety/depression		1.69 (1.00)			1.58 (0.79)	0.339	
No problem (1)	76	58.0		64	58.2		1.000
Problems (2–5)	55	42.0		46	41.8		
*Additional*: Cognition		1.72 (0.84)			1.69 (0.75)	0.798	
No problem (1)	63	48.1		50	45.5		0.699
Problems (2–5)	68	51.9		60	54.5		
EQ-5D Index (0–1)			0.73 (0.27)			0.77 (0.22)	0.220
EQ VAS (0–100)			70.1 (20.1)			68.8 (21.5)	0.631

### Negative association between anxiety/depression and HRQoL within time intervals

**Figure 1:**
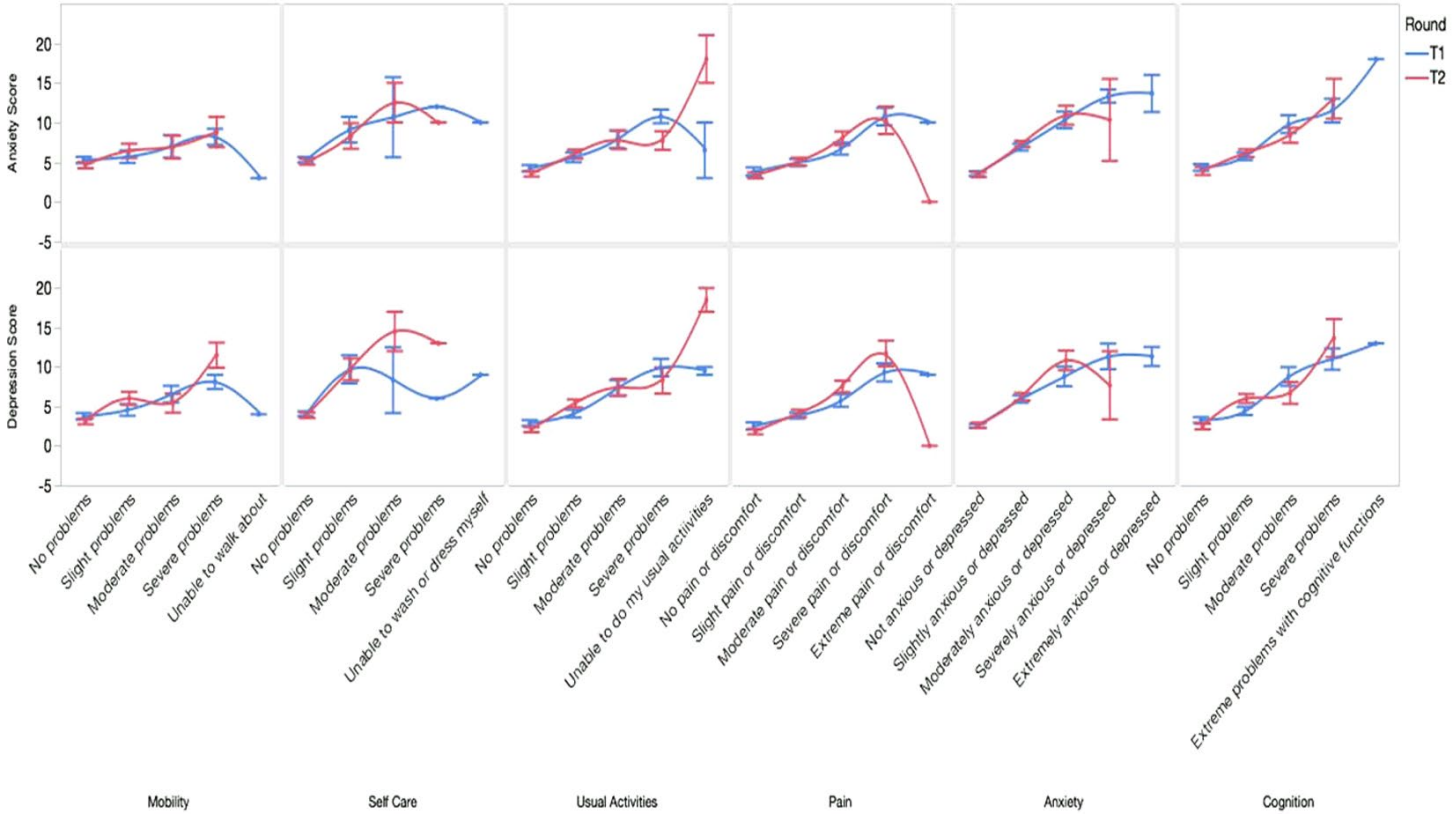
The mean anxiety and depression subscale scores and its 95% confidence interval (CI) and the level of severity of problems (from left to right: weakest to strongest problems) for each of the EQ-5D dimensions and extended cognitive dimension at T1 and T2. Note: EQ-5D, the European Quality of Life at 5 dimensions.

Fig. 1 shows that, within both time intervals, diabetes patients with a higher score in the anxiety subscale had experienced significantly more problems on all EQ-5D + C dimensions in general, i.e. mobility, self-care, usual activities, pain/discomfort and cognition (*P* < 0.05). However, respondents who had experienced extreme problems in mobility, self-care, usual activities and pain/discomfort reported fewer anxiety symptoms than those experienced moderate or severe problems (*P* < 0.05). The association between anxiety and all EQ-5D + C dimensions is similar to the association between depression and the EQ-5D + C dimensions within both time intervals, except for the non-linear association between depression and self-care at T1.

Regarding overall HRQoL in diabetes patients, those with more anxiety symptoms had a significantly lower overall rate at EQ VAS at both T1 (*P* < 0.01; R^2^ = 0.27) and T2 (*P* < 0.01; R^2^ = 0.35). The linear regression models using depression score to predict EQ VAS rate are similar to using anxiety as predictor (R^2^ = 0.29 at T1; R^2^ = 0.50 at T2).

### Effects of anxiety and depression on HRQoL over time, and vice versa

Table 3 shows that the EQ-5D summary index at T2 reflected the change in anxiety and depression symptoms between time intervals (B = −0.03) more than baseline anxiety (B = −0.01) and depression symptoms (B = −0.02). Both of the changes in anxiety and depression symptoms between T1 and T2, as well as depression at T1, significantly explained the EQ-5D summary index at T2 (*P* < 0.05), correcting for baseline EQ-5D summary index, patient’s demographic and diabetes characteristics in the Model 1 (R^2^ = 0.467) and Model 2 (R^2^ = 0.561). At univariate models: Patients who were living with diabetes type 1, employed, higher education background, and a majority ethnicity had significantly higher EQ-5D Index, thus better HRQoL (B > 0; *P* < 0.05). However, none of those characteristics were significantly associated with EQ-5D index in the multivariable models (*P* > 0.05).

**Table 3:** Linear models of anxiety/depression at T1 and change in anxiety/depression over time predicting the EQ-5D summary index at T2 (Model 1 and 2); and of the EQ-5D summary index at T1 and change in the summary index over time predicting anxiety/depression at T2 (Model 3 and 4).

			Crude model		Adjusted model for baseline outcome ^*^		Adjusted model for baseline outcome and confounders ^#^		Adjusted R^2^
			Β (SE)	*P*	Β (SE)	*P*	Β (SE)	*P*	
**Outcome: EQ-5D Index at T2**									
Model 1	Predictor	Anxiety at T1	−0.03 (0.01)	<0.001	0.00 (0.01)	0.949	−0.01 (0.01)	0.121	0.467
		Change in Anxiety	−0.01 (0.01)	0.132	−0.02 (0.01)	<0.001	−0.03 (0.01)	<0.001	
Model 2	Predictor	Depression at T1	−0.03 (0.01)	<0.001	0.00 (0.01)	0.477	−0.02 (0.01)	0.025	0.561
		Change in Depression	−0.02 (0.01)	0.014	−0.02 (0.00)	<0.001	−0.03 (0.01)	<0.001	
**Outcome: Anxiety at T2**									
Model 3	Predictor	EQ-5D Index at T1	−9.49 (1.35)	<0.001	−1.55 (1.39)	0.263	−3.14 (1.74)	0.072	0.707
		Change in EQ-5D Index	−0.04 (2.04)	0.986	−4.78 (1.36)	<0.001	−6.50 (1.46)	<0.001	
**Outcome: Depression at T2**									
Model 4	Predictor	EQ-5D Index at T1	−12.4 (1.19)	<0.001	−6.65 (1.64)	<0.001	−7.43 (1.87)	<0.001	0.720
		Change in EQ-5D Index	0.52 (2.12)	0.807	−4.07 (1.51)	0.007	−7.24 (1.43)	<0.001	

Note: B, standardized coefficient; SE, standard error; EQ-5D, the European Quality of Life at five dimensions; Higher score on EQ-5D index indicate better quality of life in mobility, self-care, usual activities, pain/discomfort and anxiety/depression, while higher scores on anxiety and depression subscales indicates more anxiety/depression symptoms. ^*^Interactions between baseline outcome and predictor were excluded in the model, as no statistically significant interactions were found (*P* > 0.05). ^#^Model adjusted for demographic and diabetes characteristics, including gender, age, diabetes type, diabetes duration, employment status, education level, ethnicity; both of the baseline predictor and change in predictor were included in the model.

Similarly, the change in EQ-5D summary index between T1 and T2 significantly explained the anxiety and depression scores at T2 (*P* < 0.05), correcting for baseline anxiety and depression scores, patient’s demographic and diabetes characteristics in the Model 3 (R^2^ = 0.707) and Model 4 (R^2^ = 0.720). The anxiety score at T2 significantly reflected the change in EQ-5D index between time intervals (B = −6.50) more than baseline EQ-5D index (B = −3.14), while depression at T2 reflects the change in HRQoL (B = −7.24) as much as HRQoL at T1 (B = −7.43). Patients who were employed and living with a majority ethnicity were less likely to have anxiety symptoms, while those who were employed, with lower education background, and a majority ethnicity were less likely to have depression symptoms (B < 0; *P* < 0.05); However, none of those characteristics were significantly associated with anxiety and depression scores in the multivariable models (*P* > 0.05).

### Longitudinal association between anxiety/depression and HRQoL with directions

**Figure 2:**
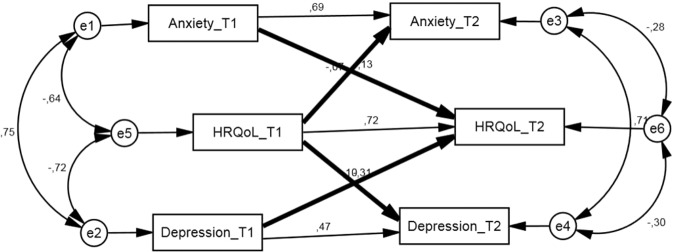
The cross-lagged model hypothesized to analyze the longitudinal association among anxiety, depression and health-related quality of life (HRQoL) summary index. Note: HRQoL is measured by EQ-5D summary index with higher score on EQ-5D index indicating better quality of life in mobility, self-care, usual activities, pain/discomfort and anxiety/depression; Numbers above arrows indicate standardized correlation coefficients; Thick arrows indicate the paths of interest, which are the bidirectional, longitudinal paths between anxiety/depression and HRQoL. The overall model fit was x^2^/df = 1.102; CFI = 1.000, RMSEA = 0.030.

We fitted our longitudinal data in a cross-lagged model with the hypothesis that there are bi-directional pathways of negative associations between anxiety symptoms and HRQoL, as well as depression symptoms and HRQoL, among adult patients with diabetes (Fig. 2). This model reached the standard of acceptable fitness (x^2^/df = 1.102, *P* = 0.256; CFI = 1.000, RMSEA = 0.030). Statistical significance was found in all the path coefficients (*P* < 0.05). Overall at T2, 46.9% of the variance in anxiety, 65.8% of the variance in depression and 86.0% of the variance in HRQoL were explained by the model.

## Discussion

This longitudinal study points at bi-directional negative associations between anxiety as well as depression symptoms and health-related quality of life (HRQoL) among adult patients with diabetes. Despite receiving diabetes care, our results during follow-up of the patients suggest direct effects of changes in anxiety and depression symptoms on HRQoL over time, as well as a direct effect of changes in HRQoL on anxiety and depression symptoms. In addition, this study shows that symptoms of anxiety and depression are common in diabetes patients, and HRQoL is suboptimal in general for patients with diabetes.

Anxiety and depression symptoms prevalence rates found in this study (27.5% and 19.8%) are almost twice as low compared to those found in another recent longitudinal study by Tonje and colleagues (47% and 38%)^30^. Both used the same instrument and thresholds to measure anxiety and depression; however, Tonje’s study included patients living with both diabetes and gastroparesis symptoms in Norway, which may explain the discrepancy. Our identified prevalence rate of depression symptoms (19.8%) is very similar to the finding of a previous review on epidemiology of depression in people with type 2 diabetes (19.1%)^31^. Also, we found that over half of the diabetes patients who had anxiety symptoms lived also with depression symptoms. This result is expected as symptoms of depression and anxiety often overlap and anxiety-depression comorbidity has been found among various populations^32,33^.

Unemployed patients with diabetes of a minority ethnic background and lower HRQoL were susceptible to develop anxiety symptoms; while those who were unemployed with higher education of a minority ethnic background and lower HRQoL were subject to depression symptoms. Nevertheless, we found that the change in HRQoL between time intervals was the most significantly associated with anxiety and depression symptoms developed over time, independent of all measured social demographics and baseline anxiety/depression level. Mood may be influenced by HRQoL through anxiety and depression systems with factors like social support and support utilization, which have a proven relationship with changing HRQoL^34^ and a proven association with psychological adjustment that influences risks of anxiety and depression^35^. This emphasizes the importance of efforts to maximize HRQoL in order to prevent anxiety and/or depression in diabetes. Future efforts to prevent anxiety and depression in diabetes care should take into consideration factors influencing HRQoL. For instance, pay attention to patients with diabetes who are struggling with insulin therapy, obesity and suffer from diabetic complications, low self-efficacy and social support, which all associate with suboptimal HRQoL^36–38^.

With regard to HRQoL status itself, we identified the average index score 0.73 at baseline, which is identical to a previous study using the same instrument among Dutch patients with type 2 diabetes^36^. Similarly, the majority of the patients with diabetes in this study reported problems in daily activities, pain and cognition, which is consistent with previous empirical findings in the US^39^. The reported mean EQ VAS rating was lower than that among a general population of the Netherlands (70.1 VS 77.7)^40^. The identified suboptimal HRQoL highlights an urgent need of interventions to improve the HRQoL in our patients with diabetes. Previous studies indicated social demographics, like ethnicity^41^, age, sex and occupation^42^, as possible determinants of HRQoL. However, we found that changes in anxiety and depression, as well as baseline depression symptoms, were the most significantly associated with HRQoL over time and independent of all measured social demographics. This indicates that interventions to prevent anxiety and depression could be beneficial to improve HRQoL in diabetes care. An important way to reduce unrecognized depression and anxiety is by advocating depression screening programs in diabetes care, particularly for patients with lower socioeconomic status and patients without a previous diagnosis of major depressive disorder^43,44^. Future efforts to improve HRQoL of diabetes patients should take into account other determinants of anxiety and depression. For instance, patients who suffer from central obesity, neuropathy, peripheral vascular disease, diabetic foot disease and pill burden are at risk for depression^45^. Poor glycemic control^46^, systolic blood pressure and fasting blood triglycerides^47^ may be indicators of a combination of depressive and anxiety symptoms in diabetes patients.

An unexpected yet interesting result is that the respondents who reported extreme problems in mobility, self-care, daily activities and pain reported fewer anxiety symptoms than those experienced moderate or severe problems. A possible explanation is resilience – patients who have been suffering from diabetes with complications for a long period may know better what they are dealing with, and have developed a better capacity to navigate their way to the psychological resources that sustain their mental well-being or accepting their situation^48,49^. Another explanation may be attrition - patients with severe health problems and anxiety and/or depression have already died, thus were not recruited by this study^50^.

This study investigated the prevalence of anxiety, depression and HRQoL among a population of adults with diabetes. To the best of our knowledge, this is the first longitudinal study evaluating the bi-directional association between anxiety symptoms and HRQoL, as well as depression symptoms and HRQoL, for diabetes patients. The questionnaires we used to measure symptoms of anxiety, depression and HRQoL had been validated widely and the results are comparable globally. In addition, considering anxiety/depression is one of the dimensions of EQ-5D, we investigated the associations of HRQoL using not only the EQ-5D summary index, but also five levels of problems for all dimensions besides anxiety/depression. Although 13.7% of the respondents were lost to follow-up, no significant differences in social demographics of respondents were found between two time points. Our study has several limitations. First, due to the nature of self-reported data, our information is likely influenced by recall bias and social desirability bias^51^. Second, we did not take into account the impact of the screening program conducted between T1 and T2, given the fact that we did not find any statistically significant difference with regards to the prevalence rates of anxiety, depression and HRQoL between the measured time points. Of course, three months of follow-up is not likely to have adequate power to detect such differences^52^. Third, we tested the direct paths between anxiety, depression and HRQoL in time, without considering possible mediators like socio-demographical characteristics, diabetes type and perceived social support, for instance, that might tangle those paths^53^. Finally, we are lacking information regarding differences between our respondents and those who did not completed the survey during the recruitment of participates, thus our sample may not be representative of all adult patients with diabetes, and we cannot exclude effects regarding order or repetition of questions in repeated surveys.

Our research results provide direct evidence to support guidelines recommending mental health screening in diabetes care, and promote efforts improving HRQoL for patients with diabetes in general. An important clinical implication is that efforts to decrease anxiety and/or depression could significantly improve HRQoL of patients with diabetes and should be tested and eventually added to diabetes care. Likewise, interventions to improve HRQoL in diabetes care are able to assist in preventing patients from developing anxiety and depression during diabetes management. Adopting an evidence-based and population-tailored collaborative care model might have potential to improve both^54^. We recommend future studies to investigate the associations between anxiety/depression and HRQoL in a prospective design with a follow-up period longer than three months, and to include more potential determinants besides social demographics, such as timing of anxiety/depression onset, obesity, insulin therapy and lifestyle factors^36^, and explore their effects in marginal models (e.g. Generalized Estimating Equations) or conditional models (e.g. Generalized Linear Mixed Models)^55^ to optimize and test future preventive measures.

## Data Availability

The datasets generated during and/or analyzed during the current study are not publicly available due to the terms of consent to which the participants agreed, but are available from the corresponding author on reasonable request.
